# Dysfunction telomeres in embryonic fibroblasts and cultured *in vitro* pluripotent stem cells of *Rattus norvegicus* (Rodentia, Muridae)

**DOI:** 10.3897/CompCytogen.v13i3.34732

**Published:** 2019-07-29

**Authors:** Natalya S. Zhdanova, Evgenia A. Vaskova, Tatyana V. Karamysheva, Julia M. Minina, Nykolay B. Rubtsov, Suren M. Zakian

**Affiliations:** 1 The Federal Research Center Institute of Cytology and Genetics SB RAS, Acad. Lavrentiev Ave. 10, Novosibirsk 630090, Russia The Federal Research Center Institute of Cytology and Genetics SB RAS Novosibirsk Russia; 2 E.N. Meshalkin National medical research center, Ministry of Health of the Russian Federation, Rechkunovskaya st. 15, 630055, Novosibirsk, Russia Ministry of Health of the Russian Federation Novosibirsk Russia; 3 Institute of Chemical Biology and Fundamental Medicine SB RAS, Acad. Lavrentjeva av. 8, 630090, Novosibirsk, Russia Institute of Chemical Biology and Fundamental Medicine SB RAS Novosibirsk Russia

**Keywords:** dysfunctional telomeres, rat, embryonic fibroblasts, ESC, iPSC

## Abstract

We studied the level of spontaneous telomere dysfunction in Rattus norvegicus (Berkenhout, 1769) (Rodentia, Muridae) embryonic fibroblasts (rEFs) and in cultured in vitro rat pluripotent stem cells (rPSCs), embryonic stem cells (rESCs) and induced pluripotent stem cells (riPSCs), on early passages and after prolonged cultivation. Among studied cell lines, rESCs showed the lowest level of telomere dysfunction, while the riPSCs demonstrated an elevated level on early passages of cultivation. In cultivation, the frequency of dysfunctional telomeres has increased in all studied cell lines; this is particularly true for dysfunctional telomeres occurring in G1 stage in riPSCs. The obtained data are mainly discussed in the connection with the specific structure of the telomere regions and their influence on the differential DNA damage response in them.

## Introduction

Telomeres are specialized structures on animal chromosome ends; they preserve chromosomes from degradation and end-to-end fusions contributing to genome stability and play an important role in the maintenance of chromosome integrity. The lagging strand of telomeric DNA consists of repeat arrays of TTAGGG and are ended by G-rich overhang, which inserts into double strand telomere region forming t-loop. As a result, the chromosome ends are no longer recognized as DSBs (Double Strand Breaks). In addition, telomeres are protected from inappropriate recombination by a specialized protein complex shelterin consisting of six proteins (de Lange 2005, 2018). Telomere shortening or any DNA lesions, disruption of telomere capping or any telomere deprotection contribute to telomere dysfunction and further replicative senescence, apoptosis, frequent chromosomal rearrangements, degenerative diseases and cancer (Karlseder et al. 2002, Kaul et al. 2011, Cesare et al. 2013).

It has been shown earlier that one unrepaired DSB in a whole genome is able to direct cells towards the replicative senescence (di Leonardo et al. 1994). However, the normal human cells proved to be resistant to four spontaneous DDR+ telomeres (dysfunctional telomeres, DNA Damage Response) arose in G1 cell phase (Kaul et al. 2011). Nevertheless, we revealed 24 of such telomeres per metaphase in the well dividing primary *Sorex granarius* (Miller, 1910) (Eulipotyphla, Soricidae) fibroblasts without of hallmarks of growth crisis (Zhdanova et al. 2014). The data about the level of telomere dysfunction in other mammalian species and in different tissue including pluripotent stem cells are restricted.

Recently, it was shown that if the chromosomes are tolerant to end-to-end fusions, then DDR of these telomeres differs from DDR in the entire genome. Such dysfunctional telomeres display differential ATM (Ataxia Telangiectasia Mutated, serine/threonine protein kinase) signaling containing not phosphorylated CHK2 (Check point 2 protein) and partial telomere deprotection with sufficient TRF2 (Telomere Repair Factor 2, component of sheltrin complex) (Cesare et al. 2013, Cesare 2014). Moreover, to overcome the replication fork stagnation near the looping structures of telomeric DNA, the cells can use a special mode of DNA replication combined with reparation pathways (Sakofsky and Malkova 2017, Sobinoff and Pickett 2017). In this way, the mechanisms involved in the occurrence of spontaneous telomere dysfunction appear to depend on the unusual structure of telomeric DNA and the disturbances in telomere specific safeguard protein complex.

γ-H2AX is usually used for detection of both manifest and invisible non repaired DNA breaks and also for visualization of deprotected telomeres (Martin et al. 2014, Cesare et al. 2013). γH2AX is a phosphorylated histone H2AX, a variant of canonical H2A histone, found in all studied eukaryotes. Being the early marker of DDR γ-H2AX is rapidly accumulated around the above disturbance. It plays an important role in the recruitment of other DNA repair-related proteins and especially in the retention of repair factors after their initial involvement (Celeste et al. 2003). Colocalized with telomeric DNA in interphase and on metaphase chromosomes, γH2AX forms foci, TIFs (Telomere dysfunction-Induced Foci) and Meta-TIFs, correspondingly (Rogakou et al. 1998, Pilch et al. 2003, Pinto and Flaus 2010). To assess the level of telomere dysfunction, Meta-TIFs are suitable rather than TIFs (Kaul et al. 2011).

Here we study the level of spontaneous telomere dysfunction in *Rattus norvegicus* (Berkenhout, 1769) embryonic fibroblasts (rEFs) and in cultured *in vitro* rat pluripotent stem cells (rPSCs), embryonic stem cells (rESCs) and induced pluripotent stem cells (riPSCs), on early passages and after prolonged cultivation. Most of the metaphases of these cell lines showed dysfunctional telomeres occurring before and after DNA replication, the last prevailed. The rESCs on the early passages of cultivation showed the lowest level of telomere dysfunction, while the riPSC lines demonstrated an elevated level. The level of telomere dysfunction increased in all studied cell lines after prolonged cultivation, especially noticeable increase in the level of telomere dysfunction arisen after G1. In addition, we have revealed a preferred accumulation of such dysfunction telomeres in riPSC lines than in rESCs. Note also the significant heterogeneity of the level of spontaneous telomere dysfunction between lines in the studied groups.

## Material and Methods

### Generation of riPSC and cell cultures

Four rEF lines were obtained from *R. norvegicus* 12-day embryos of laboratory strains Wag (RNFM1 and RNFF1 lines) and Brattleboro Wag (RWF1 and RWM1 lines). Three rESC lines (RES6, RES27, and RES28) were isolated from blastocysts of different Wag rats. Three riPSC lines (NF13, QV28, and MR39) were established as clonal lines from RNFF1 on 6 passage of cultivation by transduction. Briefly, rEFs were seeded at 10^4^ cells/cm^2^ in a six-well plate. One hour before transduction, the growth medium was supplemented with 4 mg/ml hexadimethrine bromide (Polybrene, H9269; Sigma) and the virus particles diluted in culture medium were added to wells for 18 hours (Vaskova et al. 2015). Then the culture medium was replaced with a fresh medium with doxycycline (44577; Sigma) at a final concentration of 2 mg/ml. Four days later, cells were plated on a 10-cm culture dish containing inactivated by mitomycin mouse EFs and cultured in the riPSC medium (Vaskova et al. 2015). The rESC and riPSC lines were able to differentiate into derivatives of the three germ layers (Vaskova et al. 2015). The rEF lines contained 63–67 percent of diploid metaphases, rESCs – 47–72, and riPSCs – 49–55, correspondingly. Single different Rb fusions were observed only in RES26 line (data are not presented).

### Immunochemistry and Fluorescencent *in situ* hybridization (FISH)

Meta-TIFs were visualized after immunochemistry with γ-H2AX antibodies and subsequent FISH (Fluorescent *In Situ* Hybridization) procedure with probe to telomeric DNA. Preparation of metaphase spreads for experiment was performed as described in Zhdanova et al. (2014). Briefly, adherent cultures were treated with colcemid (20 ng/ml, Sigma-Aldrich) for one hour. Cell suspensions were applied to polylisine coated slides (Thermo scientific) by cytospine centrifugation (Cytocentrifuge 7620, Wescor) at 1500 rpm for 7 min after hypotonic cell treatment with 75mM KCl. Then the slides were fixed by incubation in 4% paraformaldehyde in PBS for 10 min and permeabilized in buffer containing 120mM KCl, 20mM NaCl; 10mM Tris HCl (pH 7.5) with 0.1% Triton X-100 (Sigma-Aldrich) for 15 min at RT. The Meta-TIFs were detected with primary mouse anti- γ-H2AX antibodies (dilution 1:50, Millipore) and secondary donkey anti-mouse antibodies conjugated with Cy3 (dilution 1:100; Millipore) during 1 h at 37 °C every. The slides were re-fixed with 4% paraformaldehyde in PBS for 10 min at RT to save the fluorescent signals during conventional FISH procedure. FISH with a high sensitive telomere PNA (peptide nucleic acid) probe (CCCATT)_3_ conjugated with FITC (Applied Biosystems) was performed as previously described (Zhdanova et al. 2014). Slides were counterstained with DAPI and mounted in Vectashield medium (Vector Laboratories). 45–90 metaphases were analyzed for each cell line.

### Data analysis

Microscopic analysis and image registration were carried out using an AxioPlan 2 Imaging microscope (Zeiss), equipped with filter sets No. 49 (Zeiss),SP101 FITC and 103v1 (Chroma Technology), CCD-camera (CV M300, JAI Corporation, Japan) and running ISIS5 software (METASystems GmbH). Microscopy was performed in the Microscopic Centre of The Federal Research Center Institute of Cytology and Genetics of SB RAS, Novosibirsk, Russia. Statistics were performed using the t-test in STATISTICA 10. The results are presented as mean ±SD.

## Results

**Figure 1.**
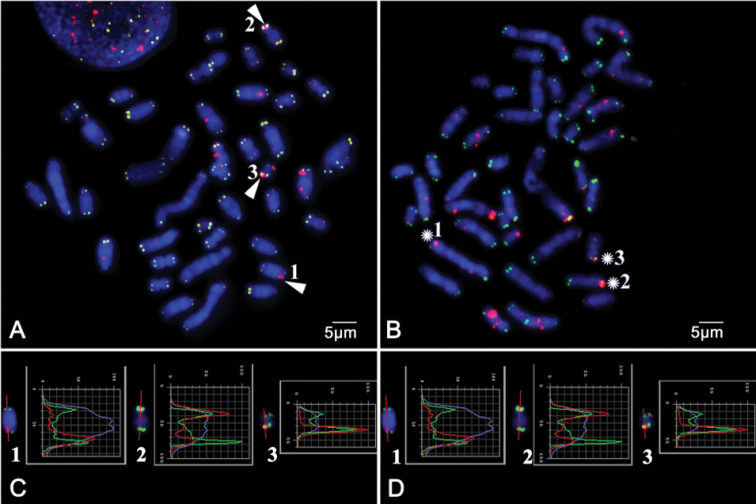
Immunochemistry with antibodies to ɤ-H2AX (red) and further FISH with telomeric PNA probe (CCCTAA)_3_ conjugated with FITC (green). Chromosomes stained DAPI (blue) **A** The metaphase spread of RNFF1 cell line on 9^th^ passage **B** The metaphase spread of MR39 cell line on 6^th^ passage. The arrows and asterisks indicate dysfunctional telomeres **C – D**. Curves of signal intensities along the length of telomeres containing dysfunctional telomeres on individual chromosomes from **A–B** respectively. Scale bars: 5 µm

The determination of *R. norvegicus* telomere status was based on the accumulation of γ-H2AX histone in telomeres of metaphase chromosomes (Meta-TIF). A fluorescent signal located on one of the two sister chromatids demonstrates the chromatid type of dysfunction (Meta-TIF of chromatid type) that has arisen after DNA replication while signals located on both sister chromatids on the one chromosomal arm confirm the chromosome type dysfunction (Meta-TIF of chromosome type) arose in prereplication period (Cesare et al. 2009, Kaul et al. 2011) (Fig. 1). For characterization of telomere dysfunction in rat lines, the following parameters were applied: number of the metaphases containing Meta-TIFs in percent; number of the metaphases containing Meta-TIFs of chromatid type in percent; number of the metaphases containing Meta-TIFs with dysfunction of chromosome type in percent; average number of Meta-TIFs per metaphase; average number of Meta-TIFs of chromosome type per metaphase and number of the metaphases carrying five and more Meta-TIFs of chromosome type in percent.

**Figure 2.**
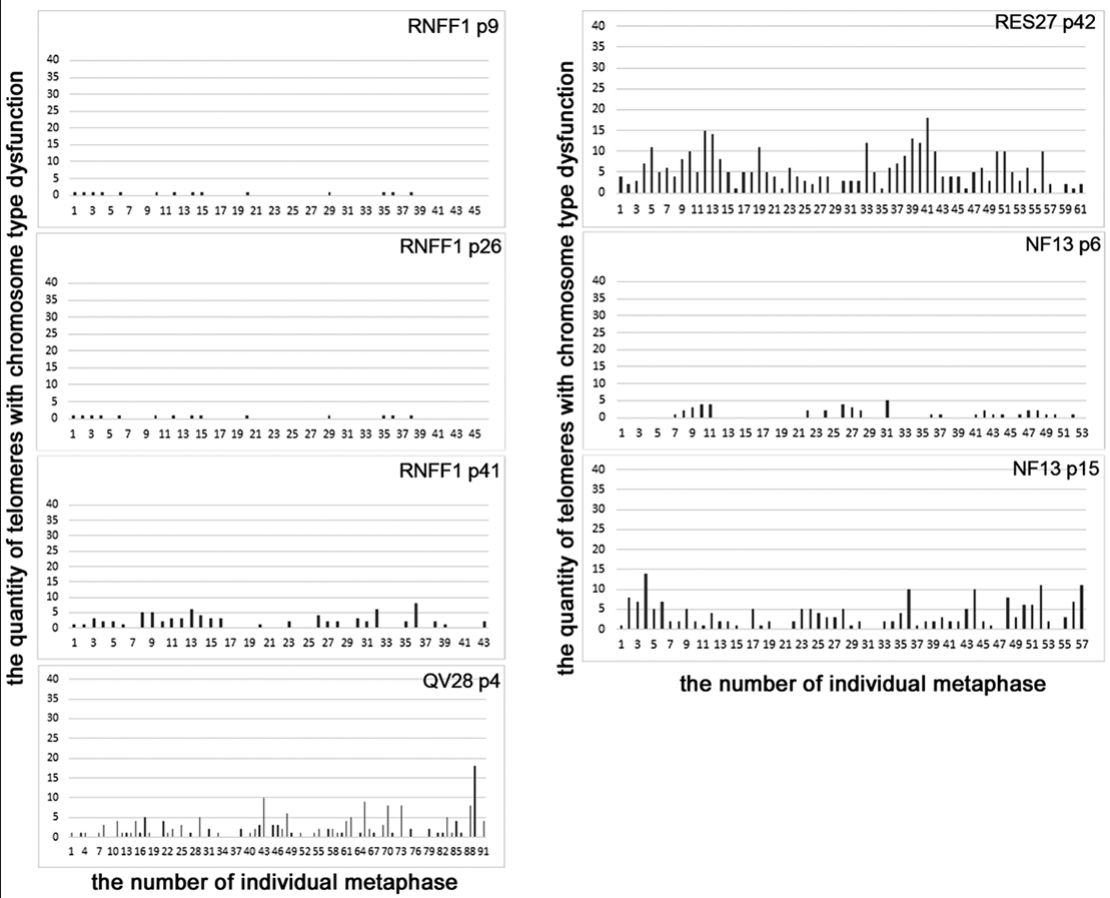
The histograms of distribution of metaphases according to a number of telomeres with chromosome type dysfunction in more interested analyzed cell lines :RNFF1 – rat embryonic fibroblasts (rEF); RES27 – rat embryonic stem cells (rESC); NF13, Q28 – induced pluripotent stem cells of rat (riPSC). P – passage. Axis x – the number of individual metaphases. Axis y – the quantity of telomeres with chromosome type dysfunction in individual metaphases.

The obtained data are presented in Table 1 and Fig. 1–2, including histograms of distribution of metaphases according to a number of Meta-TIFs with chromosome type dysfunction in the most interesting lines. Here, in the text we discussed the most informative data.

### The level of spontaneous telomere dysfunction in rat embryonic fibroblasts (rEFs)

Most of metaphases of rEFs (97 percent) on 5–9^th^ passage of cultivation contained Meta-TIFs (Table 1) with a predominance of chromatid type. 72±5.6 % of metaphases demonstrated MetaTIFs of chromatid type, while just 31±6.2 %, of chromosome type. A mean number of Meta-TIFs per metaphase was 2.76±0.77 (1.46–4.53) on average; of them, 0.40±0.13 were Meta-TIFs of chromosome type. Only 1.75±1.03 metaphases on average had five or more Meta-TIFs of chromosome type.

The rEFs grown to 23–29 passages showed an increase of almost all values used for characterization of telomere dysfunction (Table 1, Fig. 2). These lines are characterized by 6-fold an average increase in the number of metaphases with 5 and more Meta-TIFs of chromosome type (11.0±0.70 % an average). Such as RNFF1 on 9^th^ passage did not contain metaphases with five and more Meta-TIFs of chromosome type while on 26^th^ and 41^st^ passage, this line had 12 and 30 percent of such metaphases, respectively. Their maximum number was 35. In this way, during the cultivation, the rEFs tend to accumulation of dysfunctional telomeres, and especially telomeres with Meta-TIFs of chromosome type. The values relating to Meta-TIFs of chromosome type differed significantly (P<0.05) between lines on 5–9^th^ and 23–29^th^ passages, correspondingly. At the same, there observe wide disparities between studied lined in both groups on early passages and after prolonged cultivation. For instance, an average number of Met-TIFs on metaphase were 1.46 in RNFF1 on 9^th^ passage of cultivation and 4.53 in RWM1, on 5^th^ passage, and 2.66, in RWF1 on 29^th^ passage.

**Table 1.** The level of spontaneous dysfunctional (DDR+) telomeres in rat embryonic fibroblasts (rEFs) and cultured *in vitro* pluripotent stem cells of rat: embryonic stem cells (rESCs) and induced pluripotent stem cells (riPSCs).

Cell lines; passage	No. of metaphases in % with Meta-TIFs			Avr. No. of Meta-TIFs per metaphase		No. of metaphases in % with 5 and more Meta-TIFs of chromosome type
	Total	Chromatid type	Chromosome type	Total	Chromosome type	
**rEFs**						
RWF1, 6	100	57	48	3.60 (2–10)	0.68	0
RWF1, 29	100	59	71	2.66 (1–10)	1.31	12
RWM1, 5	100	72	18	4.53 (2–17)	0.07	3
RWM1, 25	72	72	65	3.83 (0–18)	1.86	9
RNFM1; 9	95	81	28	1.46 (0–8)	0.54	4
RNFM1, 23	92	72	32	3.79 (0–15)	1.05	11
RNFF1, 9	93	80	31	1.46 (0–5)	0.31	0
RNFF1, 26	86	52	65	4.81 (0–19)	1.88	12
RNFF1, 41	98	71	89	10.20 (0.30)	4.32	30
rEFs; 5–9	#97±1.80	72±5.60	*#31±6.20	2.76±0.77	*0.40±0.13	*1.75±1.03
rEFs; 23–29	87±5.90	64±2.0	*58±8.90	3.77±0.44	*1.52±0.20	*11.0±0.70
**rESCs**						
RES6; 10	92	92	29	0.90 (0–5)	0.04	1
RES27; 10	82	67	42	1.12 (0–5)	0.18	2
RES27; 42	100	41	97	14.41 (1–27)	5.47	52
RES28; 9	77	62	37	0.72 (0–3)	0.20	5
RES28; 28	96	77	84	3.55 (0–9)	0.51	9
rESCs; 9–10	#84±3.80	74±8.0	36±3.30	##0.91±0.l0	0.14±0.04	2.7±1.04
**riPSCs**						
NF13; 6	90	75	43	3.05 (0–13)	0.89	2
NF13; 15	100	56	88	12.36 (1–22)	3.59	33
QV28; 4	96	73	66	3.77 (0–15)	1.94	12
QV28; 15	96	57	69	4,96 (0–25)	2.12	13
MR39; 6	78	73	57	1.77 (0–5)	0.39	9
MR39, 22	91	67	89	7.97 (0–43)	3.17	31
riPSCs, 4–6	88±5.30	**74±0.70	**#55±6.70	##2.86±0.58	**1.07±0.46	7.7±2.97
riPSCs, 15–22	95±2.60	**60±3.50	**82±6.50	8.43±2.15	**2.96±0.43	25.7±6.37

* and ** – mark indicators differing significantly (P≥0.95) on early stages and prolonged cultivation of rEFs and riPSCs, correspondingly. # – marks indicators differing significantly (P≥0.95) on early stages of cultivation of rEFs and rESCs, rEFs and riPSCs also, correspondingly. ## – marks indicators differing significantly (P≥0.95) on early stages of cultivation of rESCs and riPSCs. The results are presented as mean ± SD.

The standard method of embryonic fibroblasts generation suggests a presence of fibroblasts at different stages of differentiation, as well as the other types of cells in primary cultures; as a rule, further cultivation leads to changes in cell content. These factors appear can affect the level of telomere dysfunction in the lines.

The level of spontaneous telomere dysfunction in rat pluripotent stem cells (rPSCs)

Similar to the rEFs, most of rESC metaphases at an early stage of cultivation contain Meta-TIFs, and Meta-TIFs of chromatid type are predominated (Table 1, Fig. 2). These lines on 9–10^th^ passages of cultivation are uniform group; and the level of telomere dysfunction in them is significantly lower than in rEFs on early passages (P<0.05). In rESCs, the average number of Meta-TIFs of chromosome type per metaphase was 0.14±0.04 an average, and 1–5 percent of metaphases contained five and more of such Meta-TIFs. During cultivation, the number of Meta-TIFs on metaphase has increased. RES28 showed 9 percent metaphases containing five and more Meta-TIFs of chromosome type on 29^th^ passage and 5 percent on 9^th^ passage. RES27 on 42^nd^ passage contained 52 percent of such metaphases, whereas on 10^th^ passage only 2 percent. The maximum number of such Met-TIFs on metaphase was 18.

riPSCs were established from RNFF1 on 6^th^ passage of cultivation. This line had the least number of dysfunctional telomeres among the rEFs (Table 1, Fig. 2). However, contrary to both parental lines and studied here rESCs, riPSCs even at early stages of cultivation demonstrated the elevated level of Meta-TIFs and was not uniform group. In riPSCs, the average number of Meta-TIFs on the metaphases was 2.86±0.58, and 2–12 percent of metaphases had five and more Meta-TIFs of chromosome type. In parental line on 9^th^ passage, these indicators were 1.46 (0–5) and 0, correspondingly; and in rECSs, 0.91±0.10 on average and 1–5, correspondingly.

Similar to the other studied cell lines, the level of telomere dysfunction in the riPSCs has increased during cultivation. Some indicators characterized Meta-TIFs of both types differed significantly (P<0.05) in riPSCs on 4–6^th^ and 15–22^nd^ passages of cultivation (Table 1). MR39 on 22^nd^ passage contained 31 percent of metaphases with five and more Meta-TIFs of chromosome type whereas 9 percent of such metaphases were observed on 6^th^ passage of this line cultivation. NF1 looked like a MR39 while this indicator has shown little variation during cultivation of QV28. Only one indicator, the mean number of Meta-TIFs of chromosome type on metaphase, differ significantly (P<0.05) in the rESCs and the riPSCs; and one indicator, the number of metaphases with Meta-TIFs of chromosome type, was significantly higher (P<0.05) in the riPSCs, than in the rEFs. In general, the pattern of telomere dysfunction in the riPSCs is looking closer to those in the rEFs than in the rESCs.

## Discussion

The rat cells, embryonic fibroblasts and cultured *in vitro* pluripotent stem cells showed low levels of spontaneous telomere dysfunction at the early stages of cultivation. The least level of telomere dysfunction characterized the rESCs whereas the riPSCs demonstrated elevated level. This applies to both types of dysfunction, chromatid and chromosomal, but increasingly to the chromosome type. For instance, the mean number of metaphases containing five and more Meta-TIFs of chromosome type in riPSCs exceeded that in normal human cells while the mean number of such Meta-TIFs on metaphase did not (Kaul et al. 2011). The dynamics of telomere dysfunction accumulation is likely to testify about the appearance of cell population prone to accumulation of higher number of Meta-TIFs of chromosome type at early stages of riPSC cultivation, and their number is increases with further cultivation. On 15–22^nd^ passage, already quarter of riPSC metaphases contained five and more Meta-TIFs with dysfunction of chromosome type; some cells demonstrated 10 and more such telomeres.

It was reported that after reprogramming mouse/human iPSCs retain some epigenetic characteristics of donor tissue and accumulate errors of DNA methylation during the reprogramming process. Transcriptome profiles vary in iPSCs obtained not only from cells of different fibroblast lines but also in genetically matched cells. In practice, iPSCs generated from different cells of heterogeneous primary embryonic fibroblasts may have significant differences, both functional and molecular (Kim et al. 2010, Wang et al. 2012, Schuster et al. 2015, Ivanov et al. 2016, Kim and Costello 2017, Noguchi et al. 2017). We observed similar trends in the level of spontaneous telomere dysfunction in distinct riPSC lines.

The comparison of iPSCs with ESCs, which is considered the ideal for *in vitro* pluripotency, shows small but distinctive dissimilarities between them in transcribed genes, epigenetic landscapes, differentiation potential, and mutation load and so on. As a whole, ESCs give the highest chance for successful subsequent differentiation when compared with iPSCs. This supports the fact that iPSCs are generally less efficient in generating a high percentage of chimeras and live mice in tetraploid complementation. And even in the reprogramming process, only a fraction of colonies considered good quality iPSC (Stadtfeld et al. 2010, Bilic and Belmonte 2012). The key features of reprogramming process during generation of rPSCs and riPSCs may underlie the telomere status in them. Since the differentiating of pluripotent cells from fibroblast progenitors is a potentially transformative tool in personalized medicine, the deeper monitoring of their attributes, including the spontaneous level of telomere dysfunction, is required, prior to the clinical implementation of pluripotent stem cell-based therapy.

The nature of spontaneous telomere dysfunction is not yet fully understood, however clear that it differs from those in the whole genome. The distinctive features of telomeres, composition of and amount of telomeric DNA and also the presence of special safeguard protein complex, result in the special mode of telomere replication and reparation of telomere DSB and other lesions. G-quadruplexes and other looping structures are in telomeric DNA in abundance (Gilson and Geli 2007). To overcome the replication stress arising after the replication fork stagnation near them, telomeres use the reparation pathways including homologous recombination and break-induced replication (BIR) in addition to the conventional mechanism of DNA replication with the help of Okazaki fragments (Sakofsky and Malkova 2017). The use of reparation pathways in the replication invariably has led to increased level of spontaneous telomere dysfunction. Sobinoff and Pickett (2017) named this mode of replication “a homology-directed recombination-dependent replication pathway that utilizes telomeric templates for synthesis”. The utilization of this mode in the case of ALT (Alternative Telomere Lengthening) activity (Cesare and Reddel 2010) considered in details (Sobinoff and Pickett 2017). The main feature of ALT cells is a recombinogenic large heterogenic telomeres (Henson and Reddel 2010). Normal mammalian cells are capable also to both intra- and extrachromosomal recombination using a mechanism similar to the use of ALT activity (Newmann et al. 2013, Dilley et al. 2016, Sakofsky and Malkova 2017) especially if they contain elongated telomeres as in ALT cells We observed unusual pattern of replication of large *S. granarius* (Miller, 1910) telomeres, containing 213 kb of telomeric repeats on average (Zhdanova et al. 2005, Minina et al. 2018).

Most mammal species are known to have telomeres less than 20 kb in length (Gomes et al. 2011). We previously described one of shrew species *S. granarius* with large recombinogenic telomeres, showing a high level of spontaneous telomere dysfunction (Zhdanova et al. 2005, 2014); wherein in the whole genome, the single DDRs are observed only in the regions of Robertsonian fusions. The rat is also a species with large telomeres, about 40 kb in fibroblasts of adult animals and about 100 kb in embryonic fibroblasts (Cherif et al. 2003, Gomes et al. 2011). It has been shown that small mammalian species with large telomeres do not use replicative senescence, and their fibroblasts can divide continuously *in vitro* (Gomes et al. 2011). We observed the nonstop division of rat embryonic fibroblast *in vitro* for half year and did not see the features of growth crisis after division of primary *S. granarius* fibroblasts for two years (Zhdanova et al. 2014). Probably, accumulation of dysfunctional telomeres in cultured primary rat and *S. granarius* fibroblasts does not lead to growth crisis and correlate with elongated telomeres.

A special protein complex shelterin protects telomeres from inappropriate recombination (de Lange 2009). In this regard, the telomere state model was developed, assuming the telomeres in protected and in a few distinct deprotected states implemented to telomere control of cellular proliferation (Karlseder et al. 2002, Cesare et al. 2009, 2013, Kaul et al. 2011). It is assumed that the induction of any alterations in shelterin components or deprotection affects the telomere dysfunction. It has been shown that a partial deprotected states activate differential ATM signaling with dephosphorylated Chk2, which do not activates G2-M checkpoint and also NHEJ pathway and end-to-end chromosome fusion, if such deprotected telomeres retain sufficient TRF2 (Telomere Repeat binding Factor 2, one of six components of shelterin). However, such telomeres activate DDR signaling and recruit ɤ-H2X (Kaul et al. 2011, Cesare 2014). If TRF2 deficit takes place, the activation of NHEJ, chromosome ends fusion with the following genome instability, a cell growth crisis, and cell death occur. Unlike whole genome, only five arisen in G1 partially deprotected telomeres with DDR signaling are able to induce replicative senescence in human cells and a more such telomeres can be accumulated without impact on growth cells in the absence of p53 signaling (Kaul et al. 2011, Cesare 2014). The telomere lengthening may contribute to decreasing of shelterin component density, including TRF2. Since ALT cells have larger telomeres and, as a rule, inactive p53 the partially deprotected telomeres may be involved in the occurrence of telomere dysfunction in them (Karlseder et al. 2002, Gocha et al. 2013, Cesare 2014) Possibly, the large telomeres at normal mammalian cells may also demonstrate the decreasing level of shelterin component density with the following resistance to elevated level of telomere dysfunction. In addition, the ability of telomere DDR signaling to persist through mitosis irrespective of telomerase activity in cells has been observed. (Hewitt et al. 2012, Fumagalli et al. 2013). These features of telometic DNA may play a role in the accumulation of dysfunctional telomeres in dividing cells as we see in the rat cells.

Spontaneous telomere dysfunction or telomere signaling are associated with the special mode of telomeric DNA replication and with a functioning of telomere safeguard complex shelterin, contributing to repression of NHEJ activation to avoid a numerous chromosomal rearrangements. They are involved in regulation of chromosome length and maintaining of genome stability.

## Conclusion

Compared with rEFs and riPSCs, the rESCs showed a reduced frequency of spontaneous telomere dysfunction on early passages of cultivation while the riPSC lines demonstrated an elevated level; moreover, the level of dysfunction was very different in studied riPSC lines. As far as cultivation, the number of dysfunctional telomeres has increased in cells of all lines; this is especially true for riPSCs. riPSCs are a model system, the study of which shows how important in personalized medicine the deeper monitoring of human iPSC attributes including the level of spontaneous telomere dysfunction before clinical implementation.
